# First anticoccidial sensitivity test applied to *Eimeria lata*: evidence of reduced sensitivity to nicarbazin in a Brazilian field isolate

**DOI:** 10.1007/s11259-026-11480-1

**Published:** 2026-08-25

**Authors:** Giovanna Fernandes Esteves, Bruno Ferraz Itoyama, Brayan Kurahara, Mariana Pavão Saraiva da Silva, Lucas Mapeli Ribeiro, Manoel Garcia Neto, Marcelo Vasconcelos Meireles

**Affiliations:** School of Veterinary Medicine, São Paulo State University (UNESP), Rua Clóvis Pestana, 793, Bairro Dona Amélia, 16050-680 Araçatuba, SP Brazil

**Keywords:** Avian coccidia, Anticoccidial drugs, Drug resistance, Poultry coccidiosis

## Abstract

*Eimeria lata* is a recently characterized species of domestic chicken whose sensitivity to anticoccidial drugs remains largely unknown. This study aimed to determine the anticoccidial sensitivity profile of the Brazilian *E. lata* BR-AMC isolate, obtained from alternative production systems, through an anticoccidial sensitivity test (AST). One hundred ninety-two broiler chicks were allocated into six treatment groups: unmedicated, non-challenged; unmedicated, challenged; and challenged groups medicated with nicarbazin (100 ppm), salinomycin (72 ppm), lasalocid (75 ppm), or semduramicin (22.5 ppm). Birds were orally challenged with 20,000 sporulated oocysts. Sensitivity was assessed using three complementary parameters: oocyst output reduction, the percentage of optimum anticoccidial activity (POAA), and body weight gain (BWG). All tested drugs reduced oocyst output by more than 80%, indicating sensitivity to all of them by this parameter. Nevertheless, based on POAA, *E. lata* was sensitive to salinomycin (96%) and lasalocid (130%), partially sensitive to semduramicin (72%), and resistant to nicarbazin (40%). BWG results demonstrated sensitivity to lasalocid, salinomycin and semduramicin, while results for nicarbazin were inconclusive. When taken together and based on the interpretation criteria adopted in this study, these divergent results indicate reduced sensitivity to nicarbazin and sensitivity to lasalocid, salinomycin, and semduramicin, underlining the importance of multi-parameter approaches when conducting ASTs. Furthermore, although based on a single isolate, our results also raise the possibility that alternative rearing systems may harbor drug-resistant *Eimeria* strains, an epidemiological concern that warrants further investigation.

## Introduction

Coccidiosis is a major disease of poultry caused by protozoan parasites of the genus *Eimeria*, and its occurrence is associated with enteritis, reduced feed efficiency, and occasional mortality (López-Osorio et al. 2020). It is one of the most economically important diseases in commercial poultry production (Mathis et al. 2025), with significant economic losses due to lower performance, prophylaxis, and treatment costs (Blake et al. 2020). Ten species are currently recognized as infecting chickens: *Eimeria acervulina*, *E. brunetti*, *E. maxima*, *E. mitis*, *E. necatrix*, *E. praecox*, and *E. tenella*, all well documented (Vrba et al. 2010), as well as *E. lata*, *E. nagambie*, and *E. zaria*, originally described as operational taxonomic units (OTUs) (Cantacessi et al. 2008) and subsequently characterized genetically and biologically as novel species by Blake et al. (2021).

*Eimeria lata* was first reported in the context of a persistent coccidiosis problem on vaccinated broiler-breeder farms (Morris et al. 2007), although its precise role in that outbreak remains unclear. Since then, this species has been reported in several countries (Fornace et al. 2013; Godwin and Morgan 2015; Clark et al. 2016; Jatau et al. 2016; Hinsu et al. 2018; Hauck et al. 2019; Terra et al. 2021), including Brazil, where a pure strain was isolated and morphologically characterized (Itoyama et al. 2025). Experimental infection with *E. lata* decreases body weight gain (BWG) in broilers inoculated with 10,000 oocysts per bird, indicating an intermediate level of pathogenicity (Blake et al. 2021).

As a prophylactic strategy against coccidiosis, anticoccidial drugs are administered in-feed and classified as either polyether ionophores or synthetic compounds (Noack et al. 2019). Resistance to anticoccidial drugs has been defined as “the ability of a parasite to multiply or to survive in the presence of concentrations of a drug that would normally destroy parasites of the same species or prevent their multiplication” (Chapman 1997). It is well known that continuous and intensive use of either ionophores or synthetic compounds contributes to the selection of resistant strains of *Eimeria* spp. (Chapman and Rathinam 2022), and drug-resistant strains have been reported worldwide wherever chickens are commercially reared (Tan et al. 2017; Kraieski et al. 2021; Mesa et al. 2021; Flores et al. 2022).

Raffaelli et al. (2026) reported the first anticoccidial sensitivity test (AST) for the novel species *E. zaria*, showing that it is susceptible to both salinomycin and a monensin-nicarbazin combination but is less sensitive to narasin. The sensitivity profile of *E. lata*, however, has not been investigated, leaving a significant knowledge gap for this cryptic species.

As part of the biological characterization of the Brazilian *E. lata* BR-AMC isolate (Itoyama et al. 2025), the present study aimed to determine its sensitivity profile to the main classes of anticoccidial compounds currently used to control poultry coccidiosis.

## Materials and methods

The study design was developed based on the general principles outlined by Cervantes and McDougald (2022) and Holdsworth et al. (2004).

## Parasite isolate

The *E. lata* BR-AMC isolate has been maintained at the Laboratory of Avian Pathology, São Paulo State University (UNESP), School of Veterinary Medicine, Araçatuba campus, through serial passage at regular intervals in coccidia-free chickens. To confirm inoculum purity, genomic DNA was extracted and screened by species-specific PCR for the seven classic *Eimeria* species (Vrba et al. 2010) and the three recently described cryptic species: *E. lata* (Blake et al. 2021), *E. nagambie* (Fornace et al. 2013), and *E. zaria* (Jaramillo-Ortiz et al. 2023).

## Birds, housing, and experimental design

One-day-old male Ross 308 broiler chicks were obtained from a commercial hatchery and housed in floor pens bedded with wood shavings until 10 days of age. Throughout the experiment, birds were kept in climate-controlled rooms under uniform lighting conditions with *ad libitum* access to feed and water. A basal corn–soybean starter diet (Table 1) was formulated using the Practical Feed Formulation Program of the School of Veterinary Medicine, Araçatuba (https://www.fmva.unesp.br/#!/extensao/projetos-e-iniciativas/ppfr/) and manufactured at the same institution. For medicated groups, anticoccidial drugs were incorporated into the basal diet at the recommended commercial inclusion levels.

**Table 1:** Ingredients and composition of the basal diet

Ingredient	%
Soybean meal	49.77
Corn	37.40
Soybean oil	6.46
Dicalcium phosphate	2.61
Premix¹	1.82
Limestone	1.29
NaCl	0.65

¹ Nugena Neo Max AA (Cargill, Brazil)

At 11 days of age, birds were transferred to battery cages, weighed individually, and allocated to one of six treatment groups: (1) unmedicated, non-challenged; (2) unmedicated, challenged; and four challenged groups medicated with (3) nicarbazin (Nicarmix 25™, Phibro Animal Health Corporation) at 100 ppm; (4) salinomycin (Coxistac™, Phibro Animal Health Corporation) at 72 ppm; (5) lasalocid (Avatec™, Zoetis Inc.) at 75 ppm; and (6) semduramicin (Aviax™ 5%, Phibro Animal Health Corporation) at 22.5 ppm. Drug doses were selected according to the recommendations established in the manufacturers’ technical product bulletins, registered with the Brazilian Ministry of Agriculture and Livestock (MAPA). At 12 days of age, groups 3–6 received their corresponding medicated diets, whereas groups 1 and 2 remained on the basal diet throughout the study.

A total of 192 birds were used, with six treatments, eight replicates per treatment, and four birds per replicate. Treatments were assigned to cages using a restricted randomization procedure to ensure a balanced distribution among battery tiers and room sides while maintaining random allocation within these constraints. Initial cage weights were similar across replicates within treatments (SD < 1 g).

The birds were monitored for oocyst shedding at the moment of housing and before challenge and remained negative at these time points. 

## *Eimeria* challenge

At 14 days of age, birds were inoculated directly into the crop using a ball-tipped gavage syringe. Birds in group 1 received 1 mL of sterile distilled water as a sham inoculation, while birds in groups 2–6 were challenged with 20,000 sporulated oocysts of *E. lata* suspended in 1 mL of sterile distilled water. This challenge dose was selected based on a preliminary dose-titration study using the same isolate. Twenty-four Ross 308 chickens were allocated to three groups of eight birds each, with two replicates of four birds per group: an unchallenged control and two challenged groups inoculated with 15,000 or 30,000 *E. lata* oocysts. Birds were weighed and challenged at 14 days of age, and final body weight was recorded 9 days post-challenge, at 23 days of age. Mean body weight at 14 days of age was 429.5, 429.25, and 428.75 g for the unchallenged control, 15,000-oocyst, and 30,000-oocyst groups, respectively, and did not differ between groups (*p* = 0.996). Mean body weight at 23 days of age was 1,157.5, 1,078.5, and 1,080.25 g, respectively, and body weight gain differed between the challenged groups and the unchallenged control (*p* = 0.003), corresponding to BWG reductions of 10.85% (15,000 oocysts) and 10.51% (30,000 oocysts) relative to the unchallenged control. This reduction is consistent with the moderate pathogenicity previously reported for *E. lata* (Blake et al. 2021).

## Assessment of anticoccidial sensitivity

The sensitivity of *E. lata* to the evaluated anticoccidial drugs was assessed using three complementary approaches. The first consisted of calculating the percentage reduction in total oocyst output compared to the unmedicated, challenged group, as described by Trujillo-Peralta et al. (2023). The second employed the percentage of optimum anticoccidial activity (POAA) index (Rathinam and Chapman 2009). The third comprised the statistical analysis of BWG, used as an indirect parameter of anticoccidial sensitivity (Chapman 1980, 1998).

## Oocyst output

To assess oocyst output, total fecal material was collected per replicate from 19 to 23 days of age (5 to 9 days post-challenge). For each treatment (1–6), feces from all replicates were pooled, placed in a beaker, and homogenized with 3,000 mL of distilled water using a benchtop mechanical stirrer. Five 1-mL aliquots of each suspension were transferred into 50-mL conical tubes, filled with 45 mL of saturated sodium chloride solution, homogenized, and loaded onto both sides of a McMaster counting chamber. Total oocyst output per 24 h for each treatment was calculated as described by Long et al. (1976). *Eimeria lata* was classified as sensitive (≥ 80% reduction), partially sensitive (31–79% reduction), or resistant (≤ 30% reduction) to each drug based on the percentage reduction in total oocyst output relative to the unmedicated challenged group (Trujillo-Peralta et al. 2023).

## Percentage of optimum anticoccidial activity

The POAA index was calculated from the growth and survival ratio (GSR), determined for each replicate by dividing the cage weight at trial termination (23 days of age, 9 days post-challenge) by the initial cage weight (11 days of age). For each treatment, POAA = [(average GSR of the medicated, challenged group − average GSR of the unmedicated, challenged group) / (average GSR of the unmedicated, non-challenged group − average GSR of the unmedicated, challenged group)] × 100. The isolate was classified as sensitive (POAA ≥ 75%), partially sensitive (POAA 51–74%), or resistant (POAA ≤ 50%) (Rathinam and Chapman 2009).

## Body weight gain

BWG was calculated as the difference between mean body weight at 11 days of age and 23 days of age (9 days post-challenge). BWG data were analyzed using a linear mixed model (LMM) fitted by restricted maximum likelihood (REML), with cage as a random effect to account for the clustering of animals within experimental units. The fixed-effects structure included age (days), treatment, and their interaction (Age × Treatment) to allow the trajectory of BWG to differ across treatments over time. Residuals were modeled with a first-order autoregressive covariance structure (AR1) within cages to account for serial correlation between repeated measurements at days 11 and 23. Multiple comparisons between treatment means were conducted using Tukey’s HSD test at a 5% level of significance. All analyses were conducted in Jamovi version 2.7 (The Jamovi Project, 2025) using the GAMLj3 module.

The efficacy of the drugs was assessed by comparing each treatment group against two controls: the unmedicated, non-challenged group (negative control) and the unmedicated, challenged group (positive control). *Eimeria lata* was classified as sensitive when the treatment group did not differ significantly from the negative control but differed significantly from the positive control; resistant when it did not differ significantly from the positive control but differed significantly from the negative control; and partially sensitive when it differed significantly from both control groups (Chapman 1980).

## Results

### Oocyst output reduction

**Fig. 1:**
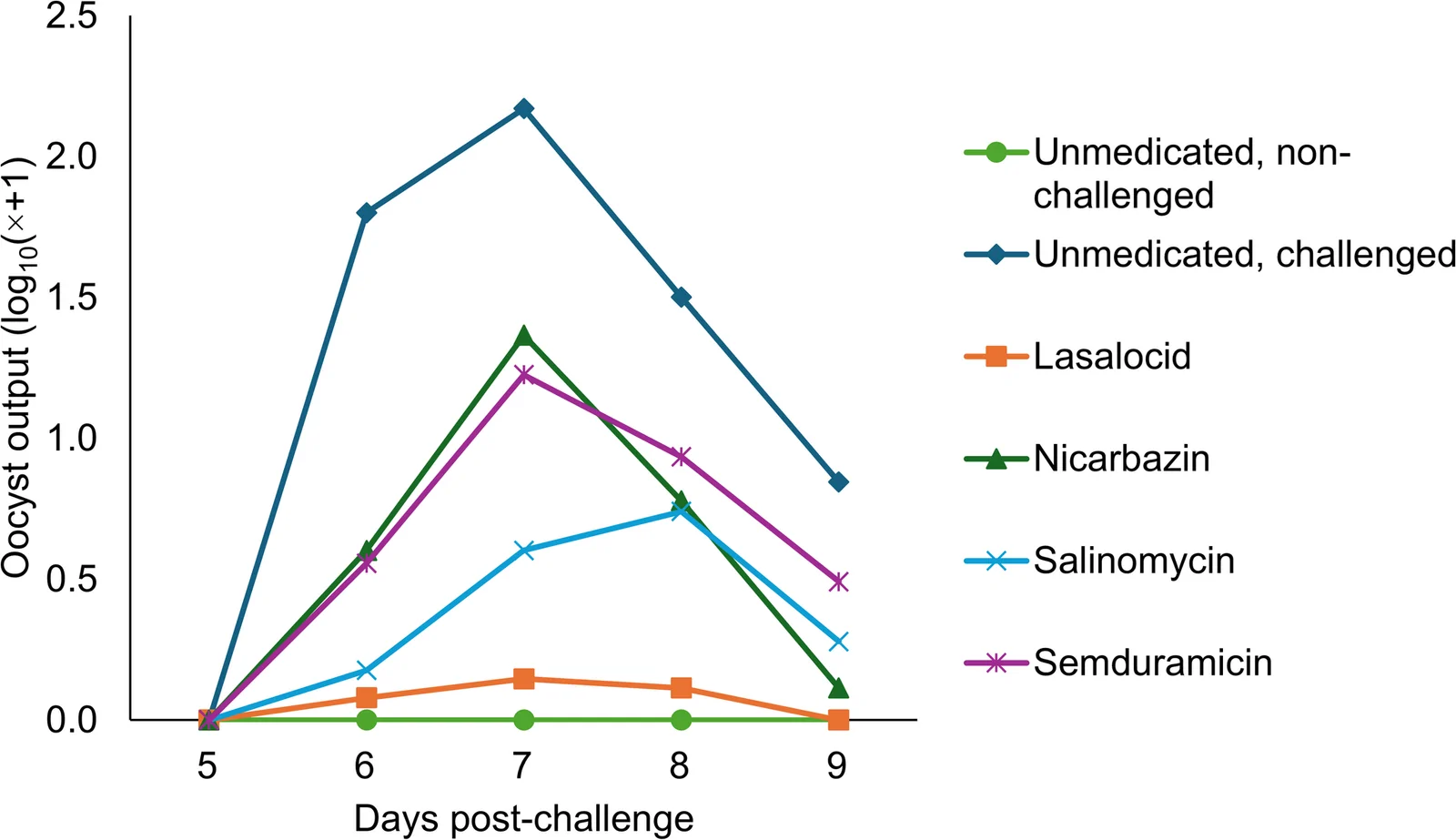
Oocyst output from days 5 to 9 post-challenge in chickens experimentally infected with the *E. lata* BR-AMC isolate and treated with lasalocid, nicarbazin, salinomycin, or semduramicin

No oocysts were found in the unmedicated, non-challenged group. Oocyst output peaked between days 6 and 7 post-challenge in all groups and declined progressively thereafter, with only a small number of oocysts being eliminated by day 9, consistent with the natural decline of oocyst shedding in the later stage of the infection cycle (Table 2; Fig. 1).

All medicated treatments achieved oocyst output reductions exceeding 80% relative to the unmedicated, challenged group (Table 2). According to the classification criteria of Trujillo-Peralta et al. (2023), *E. lata* would be considered sensitive to all four drugs based on this parameter alone.

**Table 2:** Oocyst output and percentage of reduction relative to the unmedicated, challenged group

Oocyst output (days post-challenge)	Unmedicated, challenged	Lasalocid	Nicarbazin	Salinomycin	Semduramicin
5	0	0	0	0	0
6	62.2 × 10^6^	0.2 × 10^6^	3.0 × 10^6^	0.5 × 10^6^	2.6 × 10^6^
7	147.2 × 10^6^	0.4 × 10^6^	22.2 × 10^6^	3.0 × 10^6^	15.8 × 10^6^
8	30.7 × 10^6^	0.3 × 10^6^	5.0 × 10^6^	4.5 × 10^6^	7.6 × 10^6^
9	6.0 × 10^6^	0	0.3 × 10^6^	0.9 × 10^6^	2.1 × 10^6^
Total	246 × 10^6^	0.9 × 10^6^	30.5 × 10^6^	8.9 × 10^6^	28.1 × 10^6^
Reduction (%)*	NA	99.63(S)	87.61(S)	96.38(S)	88.58(S)

*Percentage reduction in total oocyst output relative to the unmedicated challenged group (Trujillo-Peralta et al. 2023). *S* sensitive (≥ 80% reduction), *PS* partially sensitive (31–79% reduction), *R* resistant (≤ 30% reduction) (Trujillo-Peralta et al. 2023)

### Percentage of optimum anticoccidial activity and body weight gain

Analyses of the POAA showed sensitivity to salinomycin (POAA = 96%) and lasalocid (POAA = 130%), partial sensitivity to semduramicin (POAA = 72%), and resistance to nicarbazin (POAA = 40%) (Table 3).

BWG analyses using the LMM showed that the model explained 99.7% of the total variance (R² conditional = 0.997), with fixed effects alone accounting for 99.7% of the variance (R² marginal = 0.997). On day 11, body weights were homogeneous across all treatments (299–301 g), indicating successful randomization at the start of the experiment. BWG was significantly affected by age (*p* < 0.001) and treatment (*p* = 0.003). Post hoc comparisons using Tukey’s HSD test showed that BWG in the salinomycin, lasalocid, and semduramicin groups differed significantly from that of the unmedicated, challenged group (*p* < 0.001; *p* < 0.001 and *p* = 0.010, respectively), but not from the unmedicated, non-challenged group. Accordingly, *E. lata* was considered sensitive to these anticoccidials. In contrast, BWG in the nicarbazin group did not differ significantly from both control groups, indicating an inconclusive result for nicarbazin (Table 3).

**Table 3:** Percentage of optimum anticoccidial activity (POAA) and mean body weight gain (BWG) per treatment

	Unmedicated, non-challenged	Unmedicated, challenged	Lasalocid	Nicarbazin	Salinomycin	Semduramicin
POAA (%)	NA	NA	130 (S)	40 (*R*)	96 (S)	72 (PS)
BWG (g)	1,062^bc^	956^a^	1,087(S)^b^	1,007(I)^ac^	1,058(S)^bc^	1,031(S)^bc^

Means followed by different superscript letters differ significantly according to Tukey’s HSD test (*p* ≤ 0.05). POAA classification (Rathinam and Chapman 2009): *R* resistant (POAA ≤ 50%), *PS* partially sensitive (POAA 51–74%), *S* sensitive (POAA ≥ 75%)

BWG classification: *S* sensitive (not significantly different from the unmedicated non-challenged group), *R* resistant (not significantly different from the unmedicated challenged group) (Chapman 1980). *I* inconclusive: weight gain not significantly different from either control group, precluding classification into any of the above categories

## Discussion

We performed an AST to investigate the susceptibility of *E. lata* to four anticoccidial drugs representing the main classes of compounds currently used to control poultry coccidiosis: nicarbazin (synthetic), salinomycin (monovalent ionophore), lasalocid (divalent ionophore), and semduramicin (glycosidic ionophore). To our knowledge, this is the first study to evaluate the drug sensitivity profile of *E. lata*, a species whose biological characterization is still ongoing (Blake et al. 2021; Itoyama et al. 2025).

The present study differed from standard AST protocols (Rathinam and Chapman 2009; Cervantes and McDougald 2022) in two methodological aspects. First, as drug administration was initiated 48 h before challenge, birds were assigned to treatments and individually weighed at 11 days of age (coinciding with their transfer to battery cages) to ensure strict weight uniformity among replicates. This approach avoided an additional weighing procedure on the day of challenge, which would have imposed unnecessary stress on birds already acclimated to their treatments. Second, final body weight was recorded at 9 days post-challenge rather than the standard 6 to 7 days, as preliminary observations indicated that oocyst shedding had not yet ceased on day 8, suggesting that the impact of infection on growth performance had not yet fully manifested.

The three complementary parameters yielded distinct efficacy classifications. Based on oocyst output reduction, *E. lata* was classified as sensitive to all four treatments. However, POAA indicated resistance to nicarbazin and partial sensitivity to semduramicin, while BWG indicated an inconclusive result for nicarbazin. Of note, treatments with lower oocyst output reduction rates, nicarbazin (87.61%) and semduramicin (88.58%), also exhibited lower BWG (1,007 and 1,031 g, respectively) and lower POAA values (40% and 72%, respectively). Conversely, salinomycin (96.38%) and lasalocid (99.63%) achieved greater reductions in oocyst output and were associated with higher BWG (1,058 and 1,087 g, respectively) and higher POAA values (96% and 130%, respectively), indicating superior overall anticoccidial efficacy. The agreement among POAA, BWG and oocyst output reduction indicates that the relatively lower oocyst output reduction seen for nicarbazin reflects reduced sensitivity, rather than a limitation of the oocyst-count method. Nicarbazin, a chemical anticoccidial, would be expected to suppress oocyst shedding almost completely in the presence of full drug sensitivity (Chapman and Rathinam 2022). Semduramicin, in turn, also had a comparatively low oocyst output reduction and a POAA value just below the sensitivity threshold, relative to the other ionophores, suggesting a possible, though not conclusive, reduction in sensitivity; however, some degree of oocyst shedding is expected in birds treated with ionophores even under full drug sensitivity, which limits the ability of oocyst counts alone to track this discrepancy (Chapman 1999; Noack et al. 2019). The high reduction rates obtained for salinomycin and lasalocid (96.38 and 99.63%, respectively) are probably the maximal efficacy of these drugs. All these results emphasize the importance of multi-parameter approaches in ASTs, especially in the absence of universal standards for sensitivity classification (Cervantes and McDougald 2022).

Both salinomycin and lasalocid were highly effective against *E. lata*, with BWG values significantly different from those of the unmedicated, challenged group (*p* ≤ 0.05), but not from the unmedicated, non-challenged group (*p* ≤ 0.05). The sensitivity of *E. lata* to both monovalent and divalent ionophores is consistent with the expected susceptibility of a strain with no prior exposure to either of these molecules, given its origin from alternative, non-commercial production systems.

Based on POAA alone, *E. lata* was classified as partially sensitive to semduramicin (POAA = 72%), a value just below the sensitive threshold of 75% defined by Rathinam and Chapman (2009). However, BWG in the semduramicin-treated group was significantly different from the unmedicated, challenged group but not from the unmedicated, non-challenged group, meeting the BWG criterion for sensitivity. Published reports of reduced field isolate sensitivity to this drug are scarce; to our knowledge, the only published report comes from southern China (Li et al. 2004). The POAA of 72% could thus be an indication of an inherent lack of sensitivity or resistance acquired through prolonged exposure to ionophores at the farms of origin (Cervantes and McDougald 2022). To differentiate between these two scenarios, further testing of additional isolates with known drug exposure histories would be needed. In contrast with nicarbazin, two of the three parameters (oocyst output reduction and BWG) indicated sensitivity, and the BR-AMC isolate was therefore considered sensitive to semduramicin.

Nicarbazin has been used in commercial broiler production since 1955 (Cuckler et al. 1955), particularly in starter feeds, and was long regarded as a safe and effective compound with minimal resistance potential (Chapman 1984). However, this view has been progressively revised, with resistance following experimental drug selection documented in *E. acervulina* (Bafundo and Jeffers 1990) and field resistance reported in both *E. acervulina* and *E. maxima* from commercial Brazilian flocks (Kraieski et al. 2021). While oocyst output reduction classified the isolate as sensitive, POAA indicated resistance (40%) and BWG did not allow a definitive classification. Taken together, these results indicate reduced sensitivity of the BR-AMC isolate to nicarbazin.

This finding is particularly noteworthy in its epidemiological context. The BR-AMC isolate was recovered from a pooled sample collected from four farms operating under alternative production systems, where drug-resistant strains would not be expected. However, investigation of farm management practices revealed that one of the four farms had continuously administered a nicarbazin-containing product for more than 18 months prior to sampling. Because the isolate was obtained from a pooled sample, it is not possible to attribute the reduced sensitivity to nicarbazin to any individual farm. However, the farm with a history of prolonged nicarbazin use is the most plausible source, as continuous exposure to anticoccidial drugs is a recognized driver of resistance in *Eimeria* (Chapman 1997). This interpretation is further supported by a cross-sectional survey of commercial broiler farms conducted in the same region where *E. lata* BR-AMC was isolated (Dumalakas et al. 2026), in which *E. lata* was not detected. Although this evidence is indirect, it suggests that the prolonged use of nicarbazin on this specific farm may have contributed to the selection of the reduced-susceptibility phenotype observed in the BR-AMC isolate.

The present study highlights a broader concern: alternative poultry production systems are systematically underrepresented in anticoccidial resistance surveillance programs, despite accounting for a substantial share of global poultry production. In contrast to commercial broiler production, where anticoccidial drugs are routinely administered under veterinary supervision with structured rotation or shuttle programs, alternative systems, including backyard, free-range, and small-scale operations, mainly rely on live vaccines or no coccidiosis control at all. However, when anticoccidial drugs are used in these production systems, they are often administered without veterinary oversight, rotation protocols, or resistance monitoring, which creates conditions that can favor the selection of drug-resistant *Eimeria* strains. Our results offer a possible example of this risk: an *E. lata* isolate, obtained from an alternative production system, showed reduced sensitivity to nicarbazin and, if introduced into commercial flocks, could undermine existing anticoccidial programs.

A limitation of this study was the challenge dose, which produced an approximate 10% reduction in weight gain in unmedicated infected control relative to unmedicated uninfected control, below the 15–25% recommended by WAAVP guidelines for dose-determination studies (Holdsworth et al. 2004). Although the two control groups differed significantly from each other, the smaller difference in weight gain likely reduced the statistical power to classify the nicarbazin-treated group under the criteria of Chapman (1980): its weight gain did not differ significantly from either control, precluding classification as sensitive, partially resistant, or resistant, and this parameter was therefore recorded as inconclusive for nicarbazin (Table 2). This is consistent with Chapman (1998) and McDougald (1981), who noted that the statistical end-point used to classify isolates is somewhat arbitrary and directly affects the proportion assigned to each category. A higher oocyst challenge dose would likely have allowed a definitive classification of BWG results.

In conclusion, this study provides the first evaluation of anticoccidial drug sensitivity in *E. lata* using three complementary parameters: oocyst output reduction, BWG, and the POAA index. Based on the interpretation criteria adopted in this study, the combined results of these parameters indicate that the *E. lata* isolate BR-AMC exhibited reduced sensitivity to nicarbazin while remaining sensitive to lasalocid, salinomycin, and semduramicin. These findings expand the current understanding of anticoccidial sensitivity in *E. lata* and highlight the need for further investigations of *Eimeria* populations from diverse production systems.

## Data Availability

The datasets generated and/or analyzed during the current study are available from the corresponding author on reasonable request.
